# The intervening role of community-based health education in reducing unmet family planning needs among women of reproductive age 15 and 49 years in Siaya County, Kenya

**DOI:** 10.11604/pamj.2025.52.89.48467

**Published:** 2025-10-31

**Authors:** Ruth Anyango Ameso, Eliphas Gitonga, Isaac Ogweno Owaka

**Affiliations:** 1 Department of Environmental and Occupational Health, Kenyatta University, Nairobi, Kenya,; 2 Department of Family Medicine, Community Health and Epidemiology, Kenyatta University, Nairobi, Kenya

**Keywords:** Community-based health education, family planning, reproductive age

## Abstract

**Introduction:**

unmet family planning needs remain a significant health challenge. In Kenya, 14% of women have an unmet need. In Siaya County unmet need is 21% among the women, and this is high. This study seeks to determine the intervening role of community health education on the reduction of unmet needs among women of reproductive age in Siaya County.

**Methods:**

the study employed a quasi-experimental design with non-randomized, geographically distinct clusters. Assignment to the intervention and control arms was based on geographic allocation to avoid contamination into an intervention group that received structured health education for six months, and a control group, which did not. Data were collected at two time points (baseline and end line). The design enabled a difference-in-differences analysis to determine changes in outcomes between the groups over time. The FANTA formula by Robert Magnani determined the sample size of 1,448 respondents for the study. The WHO 30 by 30 two-stage cluster sampling method was used to sample the number of women of reproductive age. Data analysis was done using IBM SPSS version 28.0, with both bivariate and multivariate analyses conducted. Unmet needs for family planning were modeled using a generalized linear mixed-effects model (GLMM).

**Results:**

one thousand four hundred and forty-seven (1447) women of reproductive age (WRA) were interviewed at baseline and end line. There was a 17.1% increase in high family planning (FP) knowledge and a 12% rise in positive attitudes in the intervention, and a decline in the control group. Despite an increase in unmet need for FP in both study arms, the rise was lower in the intervention (6.7%) compared to the counterfactual (20.8%). The intervention had a protective effect against worsening of unmet need (aOR=0.31, 95% CI=0.10-1.00; p=0.051). This effect had borderline statistical significance (p=0.051). Family planning (FP) uptake decreased in the control group by 11.3% but increased in the intervention group by 6.6%, with aOR=2.42, 95% CI=0.92-6.40, p=0.075 indicating marginal statistical significance (p=0.075).

**Conclusion:**

the intervention improves knowledge and attitudes, mitigates worsening of unmet FP needs, and promotes FP uptake.

## Introduction

The use of modern contraceptive methods has been identified as critical in the attainment of the Sustainable Development Goals (SDGs) by improving women´s health. This goal is defined by SDG 3, which seeks to ensure healthy lives and promote the well-being of all ages. Importantly, the target 3.7 of SDG 3 seeks to ensure access to sexual and reproductive healthcare services, such as family planning health services, information, and education on reproductive health. Despite the SDGs´ commitment to point out the importance of reproductive health in sustainable development, there is a very high gap in unmet needs. This high gap of unmet needs has led to several challenges, such as unsafe abortions, high fertility rates, and an increase in sexually transmitted diseases [1].

According to Pillai *et al*. [2], unmet needs refer to the proportion of women who are unable to access birth control pills despite their willingness to use them. Recent studies have revealed that the number of women with unmet needs is high for women aged between 15 and 49 years [3]. This number is expected to increase by over 10% in the next ten years if nothing is done. This high number of unmet needs can be attributed to several reasons, such as lack of knowledge, concerns about the safety of birth pills, and the perceived side effects of birth pills. Other reasons for the high unmet needs can be religious and cultural issues, as well as the objections from men who object to the usage of birth pills among women [4]. The unmet needs are very high in rural and marginalized areas compared to the urban areas, which have some access to birth control pills.

The high levels of unmet needs have brought several unintended consequences, which have huge impacts on the socio-economic development of many countries. For instance, unmet needs pose a huge risk to mothers by exposing them to unintended pregnancies that may lead to other social issues. These unintended pregnancies may lead to unsafe abortions that have caused deaths to mothers in many developing countries. Other challenges of unmet needs may include high maternal and infant mortality rates during the broth periods [2]. Women facing unmet needs have also been forced out of school at very tender ages, which impacts their ability to live a decent life in the future. Unmet needs lead to early pregnancies, which may condemn women to suffer emotionally due to the judgment and isolation from society. As such, it is critical to understand how to improve voluntary family planning to eliminate the negative impacts of high unmet needs in women.

Globally, unmet needs remain a challenge despite the huge steps made by different stakeholders in the healthcare setting. In developing countries, for instance, many women prefer to postpone getting pregnant rather than use contraceptives [5]. This trend has led to the surge of traditional birth control techniques, while those who are affected choose to live with the consequences. Research shows that around 250 million people of reproductive age did not use contraceptives. As such, about 150 million women who do not use contraceptives face the challenge of risking the lives of mothers and their unborn children [6]. Most of these unmet needs are found in the sub-Saharan countries that face unparalleled challenges in the health sector. Besides, these countries have the highest fertility rates in the world, which makes it even more risky for the affected women. These countries face high poverty rates and limited resources, which impact the strides made in addressing the unmet need for family planning services.

In Kenya, the unmet needs have witnessed some enormous strides due to government intervention to educate the local communities on birth control. The country witnessed a commendable surge in birth control between 1990 and 2010 due to the sustained efforts by the ministry of health and non-governmental agencies. However, there is still a significant number of unmet needs, especially in the rural and marginalized communities, which have above-average unmet needs, Ahinkorah *et al*. [7] exposes that the unmet needs in Kenya vary by community and County, with the marginalized communities having the highest unmet needs, followed by rural counties. While some regions have witnessed a significant drop in the unmet needs, others have witnessed a slow decline, which is a cause of concern. For Instance, Siaya County has one of the highest unmet needs for birth control despite its strategic position in the Lake Basin, which is not considered a marginalized region. The County is predominantly composed of young people in the 15 - 29 years age bracket, which makes it a perfect case study to understand why it has a high unmet need.

This study sought to determine the intervening role of community-based health education on the reduction of unmet FP needs among WRA in Siaya County, by specifically looking at the two levels of unmet needs before and after the intervention. Also, the study looked at the knowledge levels and attitudes before and after the intervention of the community-based education to ascertain whether there were significant changes.

**Objectives:** 1) to determine the level of unmet family planning needs amongst women of reproductive age in Siaya County; 2) to assess the level of knowledge on family planning amongst women of reproductive age in Siaya County; 3) to determine attitudes towards family planning amongst women of reproductive age in Siaya County; 4) to determine the effectiveness of health education in reducing unmet family planning needs amongst women of reproductive age in Siaya County.

## Methods

**Study design:** the study employed a quasi-experimental design with non-randomized, geographically distinct clusters. Women of reproductive age (WRA) were enrolled through geographic allocation to avoid contamination into an intervention group, which received structured health education for six months, and a control group, which did not. Alego Usonga Subcounty was purposively selected as the intervention site, while Rarieda Subcounty served as the control. This approach was adopted to prevent contamination that could occur if women of reproductive age within the same community were randomized to different arms, as community members frequently share information. Although allocation was non-randomized, the two sub-counties share comparable socio-demographic and health system characteristics, thereby allowing meaningful comparison. Baseline measurements were conducted in both sites to adjust for any pre-existing differences between groups.

Data was collected at two points in time, at baseline (before the intervention) and at end line (six months after the intervention). This design allowed the application of a difference-in-differences analytical approach, comparing changes in unmet need for family planning between the intervention and control groups over time. The quasi-experimental design was appropriate because random allocation of participants to intervention and control groups was not feasible in the community setting, but the approach still enabled assessment of cause-and-effect relationships between the intervention and outcomes [8].

**Study setting:** the study was conducted in Siaya County, which is among the 47 counties in Kenya, and was previously considered part of Nyanza Province before the creation of the 47 counties that supported devolution. Siaya County neighbors three counties, Bungoma, Kisumu, and Busia counties. The population for Siaya County has grown exponentially, with statistics from the Kenyan population census showing that in 2019, the population was 993,183 residents, while the projections indicated that the population was likely to grow and reach 1,136,553 residents by 2027. The population of Siaya residents is predominantly young people between the ages of 15 and 29, with statistics indicating that in 2019 the population in this age group was 234,870 and was projected to grow up to 307,175 in the year 2017.

**Participants:** women of reproductive age from Siaya County who were sexually active, in committed relationships, or married were interviewed. However, the study excluded women who were critically ill. The County is among those with high levels of unmet family planning needs at 21%. Available data from the Ministry of Health put the figure at 67,023 women in unions who are sexually active.

**Variables:** the study variables included the independent variables and the dependent variables.

**Dependent variable:** dependent variable for the study was unmet FP needs and was dichotomized into either “unmet needs” “or “met need”, the set of questions asked on unmet needs were adopted from measure DHS evaluation filters, these questions included the WRA who desired to postpone pregnancies and were not currently using any contraceptives, WRA having mistimed or unwanted pregnancies at the time of this study.

**Independent variables:** independent variables in this study were social demographic characteristics, knowledge level, and attitudes of the WRA. The social demographic characteristics included age group, education, and occupation status. The knowledge on FP sought to determine the FP methods known to the participants, where to get the contraceptive, as well as the benefits of using the contraceptives. Sets of questions on the knowledge were aggregated, with the highest score being 7 and the lowest being 0. These scores were converted into percentages, with scores above 80% considered as having high knowledge, between 50% and 79% being considered as moderate knowledge, and those below 50 being considered as having low knowledge.

The attitude towards perceived benefits and risks of FP was measured by Likert-type questions with a scale of between 1 and 5 seeking levels of agreement with the questions, with a maximum aggregate score of 14 and a minimum of 0. These scores were converted into binary options, showing positive and negative attitudes towards FP. Negative attitudes were the lower score below an average of 11.63, and the high score was positive attitudes above the average of 11.63.

**Data sources:** data for the study were collected using structured questionnaires that were administered using Kobo Collect for the purpose of easier data management and storage. The data enumerators sat with the participants and self-administered the tool. Data collection at baseline took one month, while the exercise took three weeks at the end line.

**Sampling:** the study adopted the WHO 30 by 30 two-stage cluster sampling method to recruit women of reproductive age in the study, whereby the primary sampling units were the villages in the county, while the secondary sampling units were households with WRA. In the determination of the actual households in the wards, a stratification was done according to the County wards. A full list of the households was obtained from the County administrator. Serial numbers were assigned to the households, and a random number generator was used to generate a list of the households according to the ward stratification. Probability Proportional to size was done so that wards with the highest number of participants also had the highest number of households selected. Community health promoters helped in the identification of the households with WRA in the second stage of sampling.

The required sample size was calculated using the FANTA formula to obtain a minimum sample size of 724 women of reproductive age. The total sample of households with women of reproductive age was 362 households/women per study arm, yielding a total of 724 households/women in both intervention and control sites. A total of 724 women of reproductive age were enrolled at baseline, with 362 participants assigned to the intervention arm and 362 to the control arm. The same number of participants were followed up at the end line after six months of intervention. This yielded repeated measures (baseline and end line), resulting in 1,448 observations in total (724 x 2).

The FANTA formula is as follows:


$$n=D*\frac{{\left(Z_{1-\alpha /2}+Z_{1-\beta }\right)}^{2}*(P_{1}(1-P_{1})+P_{2}(1-P_{2}))}{{\left(P_{2}-P_{1}\right)}^{2}}$$

Where P_1_represents the initial estimation of unmet FP at 27%, P_2_ signifies the final level of unmet FP at 15% with the difference between P_1_ and P_2_ (12%) signifying the changes in the FP needs over time; α denotes the type 1 error at 0.05, β denotes the type 2 error at 0.20, whereas Z_1-α/2_ is the Z-score corresponding to the desired significance level (for α = 0.05, two-tailed, this is approximately 1.96); Z_1-β_ is the Z-score corresponding to the desired power of the test (for power = 80%, β = 0.20) this is approximately 0.84; D: design effect, which in this study is 2. Upon execution of the formula, a sample of 724 participants is obtained for participants in the control and intervention groups. In total across the two time frames, a total of 1448 observations were made.

**Statistical methods:** data was imported into SPSS after cleaning and used for conducting descriptive and inferential analysis, while Excel was used in the generation of bar graphs and pie charts. Percentages and frequencies were used in the descriptive analysis, whereas the inferential analysis was conducted through binary logistic regression, where odds ratios were used in the determination of the likelihood of having met FP needs. Pearson's Chi-square was also conducted to determine whether there was a significant association between the independent variables and the outcome. In particular, knowledge levels, attitudes, and unmet needs levels were determined as to whether they have a significant association with the unmet needs of FP. Using a multivariate binary regression analysis, the significance level was set at a critical value of 0.05, with calculated values below the critical value showing the independent variables were significant predictors of the outcome variable.

To ensure comparability, baseline data were collected simultaneously in both sites before the intervention, and the same questionnaires and trained data collectors were used across both groups. A Chi-square test of independence was conducted for socio-demographic characteristics. This statistical test assessed whether any observed differences in proportions between the intervention and control groups were statistically significant and whether the difference in outcome was due to the intervention and not simply due to the differences in the women themselves. Multivariable Logistic regression was used to control for confounders. During analysis, difference in difference analysis was used to determine the effect while accounting for time trends and group differences.

Outcomes were compared between the two arms by assessing changes from baseline to end line within each group and then comparing the magnitude of change between the intervention and control arms. This allowed us to account for initial differences and evaluate the relative effect of the community-based health education intervention.

**Modeling the effect of the intervention:** the data set for the two time points (baseline and end line) was pooled together. The WRA were sampled from the same villages in the two independent time points, and there was an assumption of high correlation for unmet needs within a given village, which is higher compared to the WRA in a different village. To account for this high correlation as a result of clustering at the village level, a more flexible model was applied to address the variability. In this case, the study fitted the village as a random effect because the sample was obtained from a list of villages that constituted the sampling frame; in this case, it was ideal for the study to fit a mixed-effect model comprising both random and fixed effects. The outcome was classified as either 1= yes and 2=no for the unmet needs, with 1 showing the needs had been met while 2 showed the needs had not been met. The GLMM is shown below:


$$\underset{\text{Log}\;\text{odds}}{\underset{︸}{LogY=1\left(\frac{\text{P}(y_{ijk})}{1-\text{P}(y_{ijk})}\right)}}=\underset{\text{Fixed}\;\;\text{effects}}{\underset{︸}{\beta_{0}+\beta_{1}t_{ij}+\beta_{2}X_{ik}+\overset{\text{Difference-in-deference}\;\text{term}}{\overset{︷}{\beta_{3}X_{ik}*t_{ij}}}+\beta_{4}P_{ijk}}}+\underset{\text{Random}\;\;\text{effects}}{\underset{︸}{b_{c0}+b_{c1}t_{ij}}}$$

**Indexing system:** P is the probability of meeting the need for FP, while 1- P is the probability of the unmet needs for FP. i (participant index) = 1, ??, 728, 725, ??, 1477; c (village index) = 1, ??, 74; j (time point index) = 0 (baseline), 1 (endline); k (intervention arm index) =0 (control), 1 (intervention).

**Ethical considerations:** for the study approval, both NACOSTI (ref: 404944) and the ethical review committee (ref: PKU/2996/12020) letters were considered very crucial at the point of data collection. Other ethical considerations included non-biasness, inclusivity, being culturally sensitive, obtaining consent before study, voluntary participation, and withdrawals in instances where the participants were not comfortable without reprimanding the participants. Confidentiality, integrity, and accessibility controls were upheld during data collection and management. The principal investigator remained open and transparent in explaining that the study posed no risks and there were no direct benefits for participating in the study while seeking the consent of the participants and in addition, for participants aged 15-17 years, written informed consent was obtained from their parent or legal guardian, and written assent was additionally obtained from the participant herself before enrolment.

## Results

**Socio-demographic characteristics among WRA in Siaya County between 15 and 49 years:** to assess the comparability of baseline and end-line characteristics between the intervention and control group, a Chi-square test of independence was conducted for socio-demographic characteristics. This statistical test assessed whether any observed differences in proportions between the intervention and control groups were statistically significant, as shown in Table 1. There were some differences between the groups in the variables. These differences are expected in a quasi-experimental design where random assignment is not applied. To account for these differences, potential confounding variables were controlled for during analysis using multivariable logistic regression. At baseline, those women aged 25 to 34 years were slightly more in the intervention group (48.2%) compared to the control group (42.1%). However, this difference was not statistically significant (p=0.256). At the Endline, women with secondary education were significantly higher (p <0.001) among the intervention group (52.6%) than in the control group (42.4%); 47.3% were aged 25 to 34 years, with a marginally significantly (p=0.056) higher proportion among the intervention group (50.5%) compared to the control group (44.0%).

**Table 1:** socio-demographic characteristics among the intervention and control groups, at baseline and end line

Variables	Baseline			End line		
	Intervention, n (%)	Control, n (%)	*p-value	Intervention, n (%)	Control, n (%)	*p-value
**Age (years)**						
15-24	93(25.5)	102(28.1)	0.256	87(23.3)	82(21.9)	0.056
25-34	176(48.2)	153(42.1)		189(50.5)	165(44.0)	
35-49	96(26.3)	108(29.8)		98(26.2)	128(34.1)	
**Level of education**						
No formal education	3(0.8)	6(1.7)	0.01	14(3.7)	0(0.0)	0.001
Primary	146(40.0)	158(43.5)		117(31.3)	137(36.5)	
Secondary	192(52.6)	154(42.4)		207(55.3)	197(52.5)	
Tertiary	24(6.6)	45(12.4)		36(9.6)	41(10.9)	
**Marital status**						
Married	313(85.8)	347(95.6)	<0.001	274(73.3)	309(82.4)	0.011
Single	43(11.8)	4(1.1)		96(25.7)	63(16.8)	
Wish not to say	9(2.5)	12(3.3)		4(1.1)	3(0.8)	
**Occupation**						
Business	82(22.5)	97(26.7)	<0.001	88(23.5)	28(7.5)	<0.001
Employed	17(4.7)	73(20.1)		42(11.2)	56(14.9)	
Unemployed	266(72.9)	193(53.2)		244(65.2)	291(77.6)	
**Number of children**						
None	43(11.8)	42(11.6)	0.393	28(7.5)	57(15.2)	<0.001
One	66(18.1)	59(16.3)		63(16.8)	66(17.2)	
Two	85(23.3)	76(20.9)		73(19.5)	109(29.1)	
Three	64(17.5)	66(18.2)		80(21.4)	74(19.7)	
Four	49(13.4)	40(11.0)		60(16.0)	38(10.1)	
Five	36(9.9)	43(11.8)		44(11.8)	20(5.3)	
Six and above	22(6.0)	37(10.2)		26(7.0)	11(2.9)	

* Significant at p <0.05 bold

**Figure 1:**
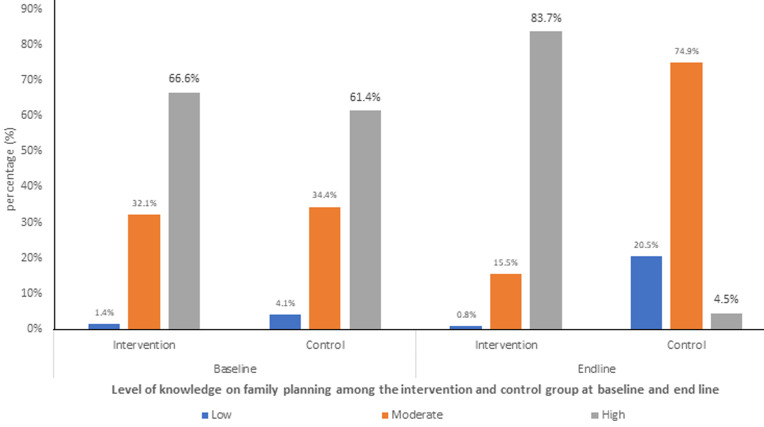
overall level of knowledge on family planning among the intervention and control group, at baseline and end line, amongst women of reproductive age between 15 and 49 years in Siaya County while seeking family planning services

**Levels of knowledge on FP amongst WRA in Siaya County at baseline (before intervention) and end line (after education):** there was a marginal increase in high knowledge by 17.1% (from 66.6% to 83.7%) in the intervention arm compared to a decrease in high knowledge in the control. At baseline, the majority of participants in both the intervention and control arms had high knowledge levels, accounting for 66.6% and 61.4% respectively. Moderate knowledge levels were reported by 32.1% in the intervention group and 34.4% in the control group, while low knowledge levels were minimal at 1.4% (intervention) and 4.1% (control), as indicated in Figure 1. At the end line, the intervention group demonstrated a marked improvement in knowledge levels, with 83.7% attaining high knowledge, an increase from 66.6% at baseline. Moderate knowledge decreased to 15.5% and low knowledge was negligible at 0.8%. In contrast, the control group experienced a decline in knowledge, with only 4.5% demonstrating high knowledge at the end line, compared to 61.4% at baseline. The majority (74.9%) of the control group exhibited moderate knowledge, while those with low knowledge increased to 20.5%.

Almost all women in the intervention group (99.7%) had received information about family planning compared to the control group (84.0%), with significant variation (p<0.001). All women in the intervention group were aware of any form of contraceptive methods compared to 88.8% in the control group (p<0.001). Similarly, all women in the intervention group were aware of the benefits of contraceptives compared to 82.9% in the control group (p<0.001). A key informant interviewed also stated: *“most of the women of reproductive age here for sure have knowledge of the available contraceptive methods in the facility. If you ask them to tell you the different types, you will be surprised that they know all of them, even male sterilization”* (Table 2).

**Table 2:** knowledge on family planning among the intervention and control groups, at baseline and end line

Variables	Baseline			End line		
	Intervention, n (%)	Control, n (%)	*p-value	Intervention, n (%)	Control, n (%)	*p-value
**Ever received any information on family planning**						
No	12(3.3)	24(6.6)	0.039	1(0.3)	60(16.0)	<0.001
Yes	353(96.7)	339(93.4)		373(99.7)	315(84.0)	
**Awareness of any family planning methods**						
No	6(1.6)	13(3.6)	0.101	0(0.0)	42(11.2)	<0.001
Yes	359(98.4)	350(96.4)		374(100.0)	333(88.8)	
**Awareness of the benefits of using contraceptives in family planning**						
No	3(0.8)	19(5.2)	0.001	0(0.0)	64(17.1)	<0.001
Yes	362(99.2)	344(94.8)		374(100.0)	311(82.9)	
**Confidence in understanding the various contraceptives for purposes of family planning**						
Not at all confident	3(0.8)	5(1.4)	0.160			
Slightly confident	38(10.4)	58(16.0)		3(0.8)	10(2.7)	<0.001
Moderately confident	77(21.1)	66(18.2)		12(3.2)	137(36.5)	
Very confident	230(63.0)	213(58.7)		46(12.3)	208(55.5)	
Extremely confident	17(4.7)	21(5.8)		313(83.7)	20(5.3)	
**Overall level of knowledge**						
Low (<50%)	5(1.4)	15(4.1)	0.047	3(0.8)	77(20.5)	<0.001
Moderate (50 -79%)	117(32.1)	125(34.4)		58(15.5)	281(74.9)	
High (80% and above)	243(66.6)	223(61.4)		313(83.7)	17(4.5)	

* Significant at p <0.05 bold

**Figure 2:**
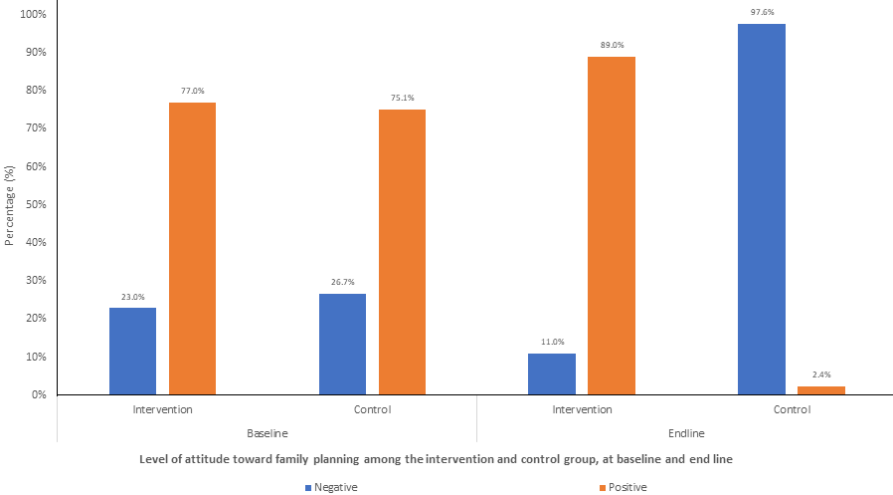
level of attitude toward family planning among the intervention and control group, at baseline and end line

**Attitude towards FP amongst WRA in Siaya County before and after intervention:** attitude was measured using a structured Likert scale ranging from strongly disagree 1 to strongly disagree 5. Positive worded statements were scored directly, while negatively worded statements were reverse-scored to ensure consistency in the interpretation. According to the study findings, there was an increase in positive attitude by 12.0% (from 77.0% to 89.0%) in the intervention arm compared to a decrease in positive attitude towards family planning by 72.7% (from 75.1% to 2.4%) in the control arm (Figure 2).

At baseline, the proportion of women who agreed that access to a variety of family planning methods is important for enabling them to make informed decisions about their reproductive health was significantly higher in the intervention group (91.0%) than control group (89.8%) (p=0.022). Women who agreed that open communication between partners is essential for building healthy spousal relationships were significantly more (p=0.005) in the intervention group (91.0%) compared to the control group (82.9%). The proportion of women who were confident about accessing and use of family planning was significantly higher in the intervention group (89.9%) compared to the control group (83.5%) (p<0.001). Women with an overall high level of attitude towards family planning were significantly (p<0.001) more in the intervention group (89.0%) than in the control group (2.4%) (Table 3).

**Table 3:** attitude towards family planning among women of reproductive age across intervention and control groups, at baseline and end line

Variables	Baseline			End line		
	Intervention, n (%)	Control, n (%)	*p-value	Intervention, n (%)	Control, n (%)	*p-value
**The importance of family planning is in controlling the size of families**						
Not important	2(0.5)	5(1.4)	0.505	0(0.0)	19(5.1)	<0.001
Somehow important	59(16.2)	56(15.4)		11(2.9)	304(81.1)	
Very important	304(83.3)	302(83.2)		363(97.1)	52(13.9)	
**Family planning is meant to benefit the well-being and health of women**						
Disagree /strongly disagree	8(2.2)	5(1.4)	0.141	6(1.6)	21(5.6)	<0.001
Neutral	26(7.1)	40(11.0)		2(0.5)	309(82.4)	
Agree /strongly agree	331(90.7)	318(87.6)		366(97.9)	45(12.0)	
**Access to a variety of family planning methods is important in helping women make decisions that are important for their reproductive health**						
Disagree / strongly disagree	6(1.6)	0(0.0)	0.022	3(0.8)	36(9.6)	<0.001
Neutral	27(7.4)	37(10.2)		5(1.3)	292(77.9)	
Agree /strongly agree	332(91.0)	326(89.8)		366(97.9)	47(12.5)	
**An open discussion between partners is crucial in fostering healthy relationships between spouses**						
Disagree /strongly disagree	9(2.5)	15(4.1)	0.005	7(1.9)	19(5.1)	<0.001
Neutral	24(6.6)	47(12.9)		17(4.5)	303(80.8)	
Agree /strongly agree	332(91.0)	301(82.9)		350(93.6)	53(14.1)	
**Support by society for family planning is more judgmental than being supportive**						
Disagree /strongly disagree	36(9.9)	45(12.4)	0.075	73(19.5)	20(5.3)	<0.001
Neutral	72(19.7)	50(13.8)		51(13.6)	293(78.1)	
Agree /strongly agree	257(70.4)	268(73.8)		250(66.8)	62(16.5)	
**I am quite confident in accessing and use of family planning when needed**						
Disagree /strongly disagree	14(3.8)	4(1.1)	<0.001	4(1.1)	18(4.8)	<0.001
Neutral	23(6.3)	56(15.4)		15(4.0)	299(79.7)	
Agree /strongly agree	328(89.9)	303(83.5)		355(94.9)	58(15.5)	
**I believe creating awareness and knowledge on family planning needs to be promoted in the community**						
Disagree /strongly disagree	2(0.5)	0(0.0)	0.364	0(0.0)	23(6.1)	<0.001
Neutral	18(4.9)	19(5.2)		1(0.3)	292(77.9)	
Agree /strongly agree	345(94.5)	344(94.8)		373(99.7)	60(16.0)	
**Overall level of attitude**						
Negative	84(23.0)	97(26.7)	0.247	41(11.0)	366(97.6)	<0.001
Positive	281(77.0)	266(75.1)		333(89.0)	9(2.4)	

* Significant at p <0.05 bold

**Unmet need of FP among WRA in Siaya County at baseline and end line for both control and intervention groups:** at the beginning of the study, a significantly larger percentage of women in the control group (51.8%) expressed the desire to have more children compared to those in the intervention group (40.5%), with a p-value of 0.002. Similarly, a higher proportion of women in the control group (32.0%) wished to delay pregnancy for at least two years but were not using any family planning method, compared to 25.2% in the intervention group (p=0.044). The use of any contraceptive method was notably higher among women in the intervention group (69.0%) than in the control group (60.6%), with a significant difference (p=0.017). Additionally, more women in the control group (20.1%) had decided not to have more children but were not using contraceptives, compared to 12.1% in the intervention group (p=0.003). The rate of pregnancy was also significantly higher in the control group (15.2%) than in the intervention group (5.2%) (p < 0.001). At the end of the study, a much higher proportion of women in the intervention group (97.1%) considered family planning very important for managing family size, compared to just 13.9% in the control group (p < 0.001). Similarly, 97.9% of women in the intervention group believed family planning benefits their health and well-being, versus 12.0% in the control group (p < 0.001). Most women acknowledged the importance of access to a range of family planning options to support informed reproductive decisions, with 97.9% in the intervention group agreeing compared to 12.5% in the control group (p < 0.001). Furthermore, the belief that open partner communication is crucial for a healthy marital relationship was much more prevalent in the intervention group (93.6%) than in the control group (14.1%) (p < 0.001) (Table 4).

**Table 4:** unmet needs of family planning among the intervention and control groups, at baseline and end line

Variables	Baseline			Endline		
	Intervention, n (%)	Control, n (%)	*p-value	Intervention, n (%)	Control, n (%)	*p-value
**Whether you currently want to have more children**						
No	217(59.5)	175(48.2)	0.002	164(43.9)	195(52.0)	0.026
Yes	148(40.5)	188(51.8)		210(56.1)	180(48.0)	
**Ever wanted to delay or avoid becoming pregnant for at least two years, but you were not using any family planning methods**						
No	273(74.8)	247(68.0)	0.044	202(54.0)	180(48.0)	0.1
Yes	92(25.2)	116(32.0)		172(46.0)	195(52.0)	
**Currently using any form of contraception to prevent pregnancy**						
No	113(31.0)	143(39.4)	0.017	90(24.1)	189(50.4)	<0.001
Yes	252(69.0)	220(60.6)		284(75.9)	186(49.6)	
**Would you like to wait at least two years before having another child, but are not currently using any contraception?**						
No	290(79.5)	275(75.8)	0.232	221(59.1)	210(56.0)	0.392
Yes	75(20.5)	88(24.2)		153(40.9)	165(44.0)	
**Unmet need for limiting- have you decided not to have any more children, but are currently not using any method to avoid pregnancy**						
No	321(87.9)	290(79.9)	0.003	285(76.2)	231(61.6)	<0.001
Yes	44(12.1)	73(20.1)		89(23.8)	144(38.4)	
**Whether currently pregnant**						
No	346(94.8)	308(84.8)	<0.001	334(89.3)	364(97.1)	<0.001
Yes	19(5.2)	55(15.2)		40(10.7)	11(2.9)	
**Prevalence of unmet need for family planning**						
Unmet need for FP	171(46.8)	213(58.7)	0.001	200(53.5)	298(79.5)	<0.001
Met the need for FP	194(53.2)	150(41.3)		174(46.5)	77(20.5)	

* Significant at p <0.05 bold; FP: family planning

Multivariable analysis for factors associated with unmet need for family planning amongst WRA age in Siaya County before and after community-based health education: a multiple regression analysis was conducted to determine the independent predictors of unmet need for family planning among women of reproductive age. Fourteen variables that showed a p-value below 0.100 in the initial bivariate analysis were selected for inclusion in the regression model. These variables included four socio-demographic factors, three related to knowledge, and seven related to attitudes. Using binary logistic regression with the backward likelihood ratio (LR) method and a removal threshold of p<0.05, the model was refined to retain five significant predictors in the final analysis, as outlined in Table 5.

**Table 5:** multivariable analysis of factors associated with the unmet need for family planning

Variables	AOR	p value
**Age (years)**		
15-24	3.43	<0.001
25-34	1.2	0.319
35-49	Ref	
**Ever received any information on family planning**		
No	3.66	0.026
Yes	Ref	
**Awareness of the benefits of using contraceptives in family planning**		
No	9.11	0.039
Yes	Ref	
**Confidence in accessing and use of family planning when needed**		
Disagree /neutral	1.89	0.011
Agree	Ref	
**Nurses are always available**		
Neutral /disagree	2.57	0.01
Agree	Ref	

AOR: adjusted odds ratio

Women in the age group of 15-24 years were found to be 3.43 times more likely to experience unmet need for family planning compared to women aged 35-49 years (adjusted odds ratio (AOR) = 3.43; 95% confidence interval (CI): 2.23-5.26; p < 0.001). Additionally, those who had never received information about family planning were approximately 3.66 times more likely to have an unmet need than their counterparts who had received such information (AOR = 3.66; 95% CI: 1.17-11.46; p = 0.026). Lack of awareness regarding the benefits of contraceptive use was also a strong predictor, with such women being 9.11 times more likely to report unmet need (AOR = 9.11; 95% CI: 1.12-74.13; p = 0.039). Furthermore, women who expressed either uncertainty or a lack of confidence in accessing and using family planning methods were 2.57 times more likely to have an unmet need compared to those who reported being confident (AOR = 2.57; 95% CI: 1.26-5.25; p = 0.010).

**Figure 3:**
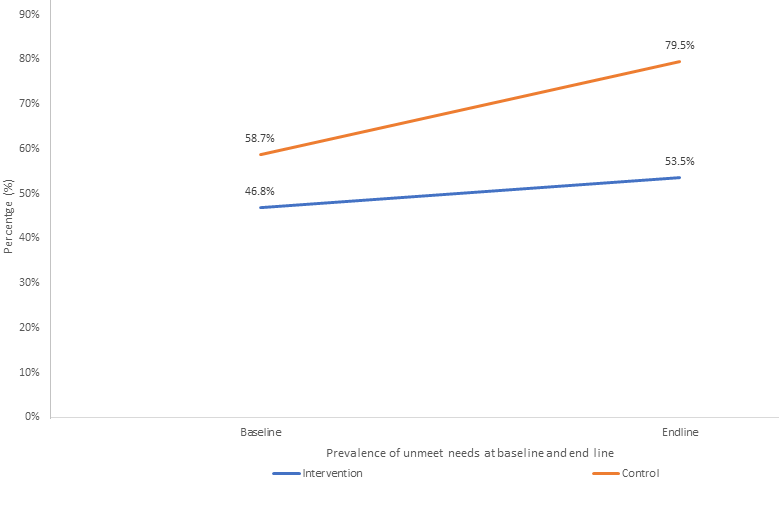
prevalence of unmet need for family planning, at baseline and end line amongst women of reproductive age in Siaya County

**Modelling of the effect of the intervention on unmet need of family planning:** to assess the impact of the intervention at the end line, data from two independently conducted quasi-experimental studies, one at baseline and the other at end line, were combined. A Chi-square test of independence was applied to examine whether differences in socio-demographic characteristics between the intervention and control groups were statistically significant. Variables that showed significance at a p-value of less than 0.05 were included in the regression model to control for any imbalances. Five socio-demographic variables were used in adjusting for the intervention's effect on unmet need for family planning: age, education level, marital status, occupation, and number of children. Unexpectedly, the level of unmet need for family planning increased in both groups. However, the rise was more pronounced in the control group, with a 20.8% increase (from 58.7% to 79.5%), compared to a more modest increase of 6.7% (from 46.8% to 53.5%) in the intervention group, as illustrated in Figure 3.

Further analysis using regression modeling revealed a significant difference in the change over time between the two groups. The adjusted odds ratio (aOR) was 0.31 (95% CI: 0.10-1.00; p=0.051), suggesting that the intervention had a protective effect. Women who participated in the intervention were 69% less likely to report having an unmet need for family planning than those in the control group. Although this effect was only marginally statistically significant, it implies potential benefits of the intervention. The borderline p-value (0.051) may reflect the limited duration of exposure, only six months to the intervention, and longer-term implementation could yield more robust improvements. Full results are provided in Table 6.

**Table 6:** effect of the intervention on unmet needs for family planning amongst women of reproductive age in Siaya County

Model	aOR (Adjusted Odds Ratio)	p-value
Intercept	1.27	0.555
Time point	3.98	0.002
Study arm: intervention	0.45	0.172
Time point*study arm: intervention	0.31	0.051
Age: 15-24	1.32	0.279
Age: 25-34	1.08	0.679
**Reference category for age in years was 35-49**		
Level of education: primary or less	1.21	0.442
Level of education: secondary	1.27	0.311
**The reference category for level of education was tertiary**		
Marital status: not married	2.04	0.002
Occupation: not employed	1.18	0.294
Number of children	0.94	0.303

* significant at p <0.05 bold

**Figure 4:**
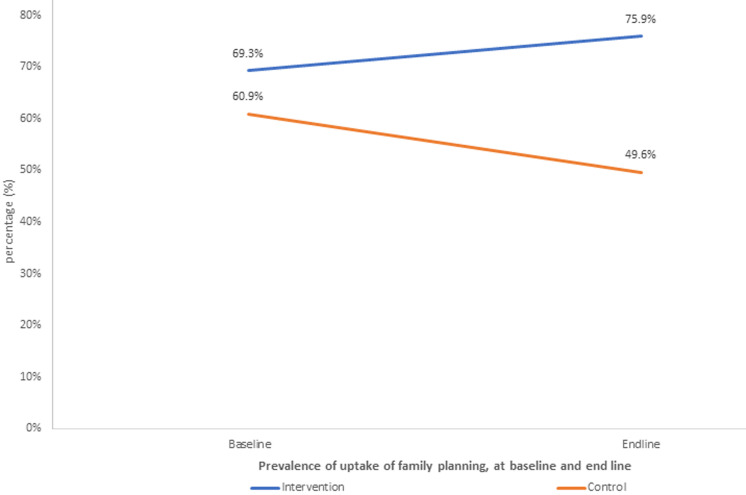
prevalence of uptake of family planning, at baseline and end line amongst women of reproductive age in Siaya County

**Modeling the effect of intervention on uptake of family planning:** similar to the unmet need for family planning, five socio-demographic factors were used in adjusting for the effect of intervention on uptake of family planning. Contrary to the anticipated change, there was a decrease in the uptake of family planning by 11.3% (from 60.9% to 49.6%) in the control arm compared to a moderate increase by 6.6% (from 69.3% to 75.9%) in the intervention arm (Figure 4).

Further modeling of the uptake of family planning revealed a significant difference in the change from baseline to end line, between the intervention and control arm (aOR=2.42, 95% CI=0.92-6.40, p=0.075). The aOR of 2.42 indicates that a participant who received the intervention was 2.42 times more likely to uptake family planning compared to one in the control arm. This effect had marginal statistical significance (p=0.075). These findings may be due to short-term exposure (of six months) to the intervention. Long-term exposure may result in a highly significant improvement in the outcome (Table 7).

**Table 7:** effect of the intervention on uptake of family planning amongst women of reproductive age in Siaya County

Model	aOR (Adjusted Odds Ratio)	p-value
Intercept	1.44	0.353
Time	0.65	0.198
Arm: intervention	1.94	0.212
Time*arm: intervention	2.42	0.075
Age: 15-24	1.14	0.575
Age: 25-34	1.62	0.006
**Reference category for age in years was 35-49**		
Level of education: primary or less	1.11	0.659
Level of education: secondary	1.03	0.896
**The reference category for level of education was tertiary**		
Married	0.43	0.000
Business/ employed	0.84	0.273
Number of children	1.01	0.798

*significant at p <0.05 bold

## Discussion

According to the study findings, the sociodemographic characteristics of participants at both baseline and end line across intervention and control groups showed differences between the groups in variables that were controlled for during analysis using multivariable logistic regression. The study findings are consistent with other quasi-experimental studies in reproductive health, Andersson *et al*. [9] conducted in Uganda, evaluating the impact of a reproductive health voucher program, and noted substantial baseline differences in education and employment among the intervention and control groups. Furthermore, Shimpuku *et al*. [10] conducted a longitudinal quasi-experimental study in rural Tanzania where they evaluated reproductive health education taught to primary and secondary school students, and noted significant baseline differences in age and the level of education among the intervention and control groups, which were controlled for in the analysis.

Additionally, according to the study findings, there was a marginal increase in high knowledge in the intervention arm compared to a decrease in high knowledge in the control arm. These findings are consistent with those of a recent quasi-experimental study conducted in Tanzania by Mushy *et al*. [11] whose respondents were pregnant adolescents who sought to evaluate the effectiveness of a decision-aid intervention for long-acting reversible contraception (LARC). The findings showed a significant increase in contraceptive knowledge scores in the intervention group at baseline compared to the control group.

Furthermore, the findings indicate that there was an increase in positive attitude in the intervention arm, compared to a decrease in positive attitude towards family planning in the control arm.

Contrary to the anticipated change, the study findings showed that there was an increase in unmet need for family planning in both study arms; a high increase in the control arm compared to a moderate increase in the intervention arm. Similarly, the study revealed a significant difference in the change from baseline to end line between the intervention and control arm. Further analysis of the odds ratio indicated a protective effect of the intervention compared to the control. This effect had borderline statistical significance. The findings are consistent with previous work by Sileo *et al*. [12] who reported that sustained community-based health education was significantly effective in bringing about long-term behavioral change regarding FP utilization and thus diminishing the unmet family planning need among women of reproductive age. According to the findings of the study, there was a decrease in uptake of family planning in the control compared to a moderate increase in the intervention arm. Further modeling of the uptake of family planning revealed a significant difference in the change from baseline to end line, between the intervention and control arm, and that a participant who received the intervention was 2.42 times more likely to uptake family planning compared to one in the control arm. This effect had marginal statistical significance. These findings agree with a similar study in study conducted in rural Uganda [12], which evaluated the impact of community health worker-led education on contraceptive use. The study findings showed a significant increase in modern contraceptive use in the intervention group compared to a marginal change in the control group. Similarly, the findings from the study showed that community-based education improved knowledge and attitude, leading to improved uptake of family planning.

**Recommendations:** the study recommends that there is a need to scale up community-based health education Interventions in order to reduce unmet needs for family planning, because despite the overall increase in unmet needs in both arms, the intervention group experienced a slight increase compared to the control group, with an adjusted odds ratio indicating a protective effect. This suggests that community-based health education can mitigate the worsening effect of unmet need. Additionally, the increase in unmet need even within the intervention group, though modest, indicates that high knowledge and positive attitude alone may not be sufficient to increase uptake of family planning and reduce unmet needs for family planning. other barriers may still hinder uptake, and therefore, community-based health education interventions should be complemented with other strategies. Also, there is a need to address other underlying barriers beyond knowledge and attitude. Finally, a longer intervention period should be implemented to increase uptake of FP and reduce unmet need for family planning.

**Limitations:** Siaya County, being a vast one, posed logistical challenges during the administration of the tool, which called for extra resources to reach participants in multiple locations. Besides, the topic of FP is an emotive one and requires participants who call for the community health workers' intervention to have the responses. Lastly, confidentiality insecurities and issues arose where participants were worried about their personal details and feedback going to the public without their permission.

## Conclusion

This study concludes that community-based health education has a protective effect in the reduction of unmet needs for family planning among WRA in Siaya County. Similarly, community-based health education can be used as a promising strategy for addressing the challenge of unmet need for family planning. Furthermore, community-based health education has an effect on the knowledge and attitude of women of reproductive age by increasing awareness, enhancing a positive attitude, and encouraging informed decision-making for contraceptive uptake.

### What is known about this topic

- The study is fully aware that, through other studies, there are very high levels of unmet needs in Siaya county, which is above the national average;
- The challenges require a multifaceted approach to address the knowledge levels and boost access to contraceptives.

### What this study adds

- We found that higher knowledge and positive attitudes towards family planning are strongly associated with reduced unmet family planning needs among women of reproductive age in Siaya County;
- We demonstrated that community-based health education delivered by trained community health promoters is effective in increasing knowledge and improving attitudes, and mitigating worsening of unmet family planning needs among women of reproductive age in Siaya County.
